# “Reassessing CT‐Verified Micra Pacing Locations: Methodological Limitations and Future Clinical Directions”

**DOI:** 10.1002/clc.70303

**Published:** 2026-04-17

**Authors:** Ibadullah Tahir, Hunain Shahbaz

**Affiliations:** ^1^ Ayub Medical College Abbottabad Pakistan

Dear Editor,

The article published recently by Zhang et al. describing a CT‐verifying Micra pacemaker placement has generated interest; its strongest component is the extensive application of a post‐implant 3D cardiac CT scan to locate and verify leadless RV pacing placement locations compared with standard fluoroscopy alone. Prior research has demonstrated that both fluoroscopic images and ECG criteria frequently incorrectly categorize septal and free wall implant positioning [1, 2]. In confirming the Micra tip position with CT, Zhang et al. confirmed that most Micra implants were located mainly within an anterior septal/free wall “hinge” region and related these specific regions to ECG pacing patterns for leadless RV pacing. These CT imaging data support prior studies and demonstrate that the placement of the lead tip (when confirmed with CT) is useful for prognostic determination [3]. The authors deserve recognition for their use of advanced imaging techniques to better understand the anatomy of leadless RV pacing.

There are some factors to consider. First, the study focused on a small sample (*n* = 20) of bradycardic patients in one centre only, so the potential for selection bias is high and the results will be limited in their generalizability. All participants had a normal baseline LVEF, and so the findings may not apply to those with LV dysfunction or structural heart disease. Second, although the participants were identified prospectively for inclusion in this study, a separate analysis was then performed to compare “septal” versus “free wall” sites of implantation. This later analysis was not prespecified and therefore is based on retrospective evidence. Furthermore, the classification of pacing locations into “septal” or “free wall” is itself somewhat subjective; as previously reported by Tsukahara et al., the septum/free wall boundary (the ‘hinge’) represents a transition zone that makes it difficult to determine the pacing location accurately using only electrocardiographic data [5].

Attributing outcome variations to these zones is problematic due to the absence of an independent gold standard or randomized assignment. The follow up period (3 months) was very short, and clinical endpoints were not reported. Since pacing induced desynchrony may have an effect on LV over several months, clinicians should be careful when interpreting the minor LVEF differences and event free status that were reported in this study. The QRS patterns of paced patients that have the same morphology as left bundle branch block have been documented to cause pacing induced cardiomyopathy later [4]. In addition, complications related to the Micra device have been documented by the EPLS in post market research [6]. Thus, there is uncertainty regarding the clinical significance of the differences between the ECGs and LVEF until longer term follow up is.

Recommendation: We encourage researchers to continue this type of research using improved methodology. Future areas of potential research could include:

1 Increased number of prospective studies with larger patient populations with CT verified Micra tip positions by following patients longitudinally for clinical endpoints including LVEF, heart failure symptoms, and major events [3] and to see if either septal vs free wall implantation would have an effect beyond procedural endpoints.
2 Imaging/ECG guidance algorithms would include the development and validation of approaches utilizing fluoroscopy, contrast angiography, and ECG based criteria to allow the clinician at the time of Micra placement to direct and correct for the appropriate septal placement. For example, calibrated angles for RAO/LAO and/or the use of ECG algorithms to obtain a true placement in the septum may be calibrated to CT verification (with correction for the hinge) in order to improve the probability of correct placement during the procedure [5].
3 Noninvasive or Intraoperative Activation Mapping and Computer Tomography as Combined Anatomical and Electrical Mapping:
  High Detailed Mapping for Ventricular Activation Clarification on the Effect of Different Micra Locations on LV Synchronous Activation: Possibly Provide Guidance on an Implantation Methodology. Steps Taken Will Provide Definitions of the Relationship Between Patient Outcome and CT Defined Pacing Location. Therefore, Zhang et al. Offer Significant Anatomical Insight but Require Future Prospective and/or Outcome Driven Study Validation Before Clinically Implementing This Information.

## Author Contributions

All authors have read and approved the final version of manuscript.

## Conflicts of Interest

The authors declare no conflicts of interest.

## Data Availability

The authors have nothing to report.
